# Simulation of Corrosion Cracking in Reinforced Concrete Based on Multi-Phase Multi-Species Electrochemical Phase Field Modeling

**DOI:** 10.3390/ma18163742

**Published:** 2025-08-10

**Authors:** Tianhao Yao, Houmin Li, Keyang Wu, Jie Chen, Zhengpeng Zhou, Yunlong Wu

**Affiliations:** 1 School of Civil Engineering, Architecture and the Environment, Hubei University of Technology, Wuhan 430068, China; 2 Wuhan Construction Engineering Group Co., Ltd., Wuhan 430014, China

**Keywords:** chloride ion intrusion, electrochemical reactions, cracking, multi-species ions, phase field

## Abstract

Non-uniform corrosion cracking in reinforced concrete buildings constitutes a fundamental difficulty resulting in durability failure. This work develops a microscopic-scale multi-species electrochemical phase field model to tackle this issue. The model comprehensively examines the spatiotemporal coupling mechanisms of the full “corrosion-rust swelling-cracking” process by integrating electrochemical reaction kinetics, multi-ion transport processes, and a unified phase field fracture theory. The model uses local corrosion current density as the primary variable to accurately measure the dynamic interactions among electrochemical processes, ion transport, and rust product precipitation. It incorporates phase field method simulations of fracture initiation and propagation in concrete, establishing a bidirectional link between rust swelling stress and crack development. Experimental validation confirms that the model’s predictions about cracking duration, crack shape, and ion concentration distribution align well with empirical data, substantiating the efficacy of local corrosion current density as an indicator of electrochemical reaction rate. Parametric studies were performed to examine the effects of interface transition zone strength, oxygen diffusion coefficient, protective layer thickness, reinforcing bar diameter, and reinforcing bar configuration on cracking patterns. This model’s multi-physics field coupling framework, influenced by dynamic corrosion current density, facilitates cross-field interactions, offering sophisticated theoretical tools and technical support for the quantitative analysis, durability evaluation, and protective design of corrosion-induced cracking in reinforced concrete structures.

## 1. Introduction

Reinforced concrete, the predominant composite material in contemporary civil engineering, is crucial in infrastructure building owing to its superior compressive strength, remarkable durability, and distinctive synergistic properties. Chlorides can infiltrate the concrete protective layer via diffusion, resulting in electrochemical corrosion of the reinforcing steel, which causes concrete cracking [1] and accelerates the degradation of reinforced concrete [2,3,4]. In maritime conditions, the predominant cause of concrete cracking is the infiltration of chloride ions. The progression from chloride intrusion to the corrosion of reinforcing steel and resulting cracking of concrete entails interactions across multiple physical fields, underscoring the critical necessity for multi-physics field research, particularly studies on electrochemical–mechanical coupling, to comprehensively examine the intricate interactions between electrochemical and mechanical mechanisms throughout the corrosion process.

Over the past few decades, numerous academics have researched the inherent mechanisms of concrete cracking caused by the corrosion of steel reinforcement through experimental investigation, theoretical analysis, and numerical simulation. Notably, Ye et al. [5] focused on corner-located rebars, a critical yet understudied scenario, and found that their rust distribution—well-characterized by Gaussian functions—exhibits distinct bimodal or unimodal patterns depending on the ratio of concrete cover thicknesses on both sides, directly influencing cracking behavior. Complementing this, Pedrosa and Andrade [6] explored the dynamic interplay between corrosion rate and crack evolution, demonstrating a bilinear growth process where the ratio of crack width to steel attack varies with corrosion intensity, thus emphasizing the need to account for rate effects in predictive models. In the domain of numerical simulation for rust boundary definition, by contrast, traditional methodologies such as the linear decay model [7,8,9], quadratic curve model [10,11], Gaussian model [12], and von Mises model [13] have been extensively utilized in the scholarly literature to mathematically characterize the spatial distribution of corrosion products. However, in simulating chloride-driven corrosion processes, these models often rely on preset rust morphologies and fail to dynamically reflect the real-time influence of chloride concentration gradients on rust non-uniformity. Such limitations impede their capacity to capture the intrinsic coupling between chloride transport kinetics and rust growth patterns. Numerical models with temporal dynamic evolution have been systematically developed and refined to overcome the technical limitations of conventional experimental and numerical analysis methods in understanding the initiation and propagation characteristics of concrete cracking. Notable advancements have been achieved in the techniques of the crack banding method [14], damage model [15], discrete cracking model [16], and extended finite element method (XFEM) [17]. While traditional numerical methods can proficiently simulate the mechanical cracking process, they encounter notable challenges: firstly, specific models reveal crack extension path dependence on meshing, severely compromising the accuracy and generalizability of simulation outcomes; secondly, there is the question of how to achieve efficient coupling of multiple physical fields within a unified computational framework.

Non-local methods can more precisely capture the intricate physical correlations and interfacial effects across regions in multi-field coupling processes due to their inherent consideration of long-range interactions between spatial points employing non-local kernel functions. For instance, non-local numerical analyses such as the phase field method [18] and peridynamics [19] are gaining prominence in the simulation of crack evolution in solid materials. At present, peridynamics has been effectively utilized in sulfate-induced cracking [20], premature concrete cracking [21], and galvanic corrosion [22]. The phase field method characterizes material damage interfaces via a scalar field, enabling it to accurately capture the initiation, propagation, and bifurcation of cracks across spatial and temporal scales. This attribute renders it particularly well-suited for multi-phase materials like concrete. The phase field cohesive crack model (PF-CZM) introduced by Wu et al. [23,24,25] amalgamates the strength criterion for crack initiation, the energy criterion for propagation, and the path selection mechanism, rendering it particularly applicable to nonhomogeneous material systems like concrete, which encompasses the aggregate–mortar interface and the transition zone. The PF-CZM is especially appropriate for heterogeneous material systems like concrete, which features an aggregate–mortar interface transition zone. The model necessitates only a limited number of material parameters to simulate damage progression under intricate stress conditions effectively [26]. Furthermore, the analytical outcomes exhibit insensitivity to mesh density and phase field scaling parameters, demonstrating the phase field method’s distinct advantage in the coupled simulation of multi-physical field behavior related to corrosion cracking in reinforced concrete.

In the multi-physics field, coupling simulation of the corrosion process of reinforced concrete under chloride ion erosion using the phase field model, relevant research has made significant progress. Through theoretical derivation and empirical validation, Wei and other researchers [27] established a quantifiable correlation between rust expansion strain and oxygen flux. It follows that the cracking mechanism and developmental pattern of concrete structures subjected to uniform corrosion were comprehensively examined utilizing the diffusion–mechanical coupled phase field model. Fang et al. [28] innovatively constructed an electrochemical–mechanical coupled phase field model to investigate the crack morphology of concrete caused by non-uniform corrosion. Subsequently, the research team further extended it to a multi-phase field model [29], which can simultaneously capture the initiation and propagation of concrete cracks and the dynamic evolution characteristics of the corrosion interface. Korec E et al. [30] developed a novel framework for the corrosion and cracking mechanisms of reinforced concrete, elucidating the chemical–mechanical coupling process, hence offering a robust theoretical foundation for research in the domain of reinforced concrete corrosion and cracking. Subsequently, the research team developed a phase field chemical–mechanical model for chloride-induced corrosion cracking [31], which accurately represents various corrosion processes initiated by the corrosion of reinforcing bars at the mesoscale, as well as the cracking behavior of concrete. This model has become an essential tool for elucidating the cross-scale evolution mechanism of reinforcing bar corrosion and concrete damage in chloride environments. Building on these advancements, a coupled electro–chemo–mechanical model [32] specifically addressing reinforced concrete corrosion and crack propagation has further refined the integration of electrochemical kinetics, ionic transport, and mechanical damage. It offers a more nuanced portrayal of how these interconnected processes drive degradation in chloride-exposed structures.

Currently, no multi-physical field coupled phase field cracking model incorporates multiple ionic interactions. Previous models have oversimplified rebar corrosion as a homogenized macroscopic process, presuming constant rebar surface reactivity. This approach neglects the intricate local coupling between corrosion microcells and concrete cracking, resulting in an inadequate characterization of the interaction mechanism between electrochemical reactions and mechanical damage dynamics. The implementation of a variable corrosion current density model effectively encapsulates the electrochemical inhomogeneity within the local anodic activation zone and its influence on crack initiation and propagation, thereby achieving a nuanced characterization of the cross-scale coupling process between corrosion and cracking, and offering a more precise analytical framework for elucidating the damage evolution mechanism of reinforced concrete structures in chloride environments.

This essay presents the development of a coupled multi-species, multi-physics mesoscale model for the numerical simulation of critical physicochemical processes, including the electrochemical reactions of oxygen with ions in concrete, rust precipitation, and concrete cracking. This model simulates the corrosion and cracking phenomena in reinforced concrete structures, with multiple coupled physical fields influenced by variable corrosion current densities. This essay’s primary contributions are as follows:

(1) Linked model of dynamically variable corrosion current density, including electrochemical reactions and mechanical fields, is created based on the phase field approach to facilitate the unified simulation of corrosion and cracking processes.
(2) A multi-phase multi-species coupled phase field model is developed at the fine-scale level, comprehensively integrating multi-species ion transport, electrochemical reactions on the steel surface, and the phase field model.
(3) Simulation of dynamic electrochemical interactions among various rebar configurations for investigating the factors influencing their cracking patterns.

## 2. Multi-Physics Field Framework

In the very alkaline environment (pH 12–13) within concrete, reinforcing steel undergoes a reaction that produces a nanoscale passivation layer, thereby safeguarding the steel.

**Figure 1:**
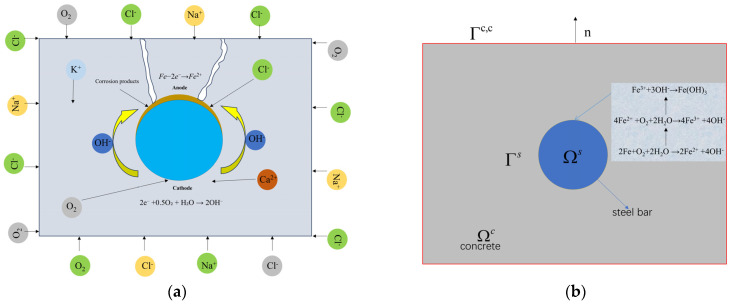
Schematic diagram of corrosion evolution of reinforced concrete: (**a**) Rebar corrosion and concrete cracking; (**b**) Graphical description of the field and related variables.

This protective process can be undermined by chloride corrosion, resulting in localized disruption to the passivation layer, as seen in Figure 1a, and initiating localized corrosion [33,34,35]. When the passivation film on the surface of the reinforcing steel is locally compromised, the anodic dissolution reaction commences: Fe → Fe^2+^ + 2e^−^ electrons. The electrons generated in this process are utilized in the cathodic reduction reaction, therefore preserving charge equilibrium: 2e^−^ + 0.5O_2_ + H_2_O → 2OH^−^. Figure 1b illustrates that the outer layer signifies the concrete boundary $\Gamma^{c,c}$, and the concrete mortar domain $\Omega^{c}$. The corrosion of chlorides disrupts passivation, resulting in a potential difference on the surface of the reinforcing bars $\Gamma^{s}$, which creates anodic and cathodic surfaces, leading to electrochemical processes that cause rusting of the reinforcing bars. In the cathode area, oxygen reacts with water to produce hydroxide ions. In the anodic region, iron corrodes to produce rust, which increasingly accumulates along the interface between the reinforcing steel and concrete. The volume expansion rate of these products may reach 2–4 times that of the original metal volume [36], resulting in rust expansion that induces cracking of the concrete protective layer under pressure, ultimately leading to failure. The equation for the reaction is as follows:

(1) $$2\mathrm{Fe}+O_{2}+2H_{2}O\to 2{\mathrm{Fe}}^{2+}+4{\mathrm{OH}}^{-}$$

(2) $$4{\mathrm{Fe}}^{2+}+O_{2}+2H_{2}O\to 4{\mathrm{Fe}}^{3+}+4{\mathrm{OH}}^{-}$$

(3) $${\mathrm{Fe}}^{3+}+3{\mathrm{OH}}^{-}\to \mathrm{Fe}{\left(\mathrm{OH}\right)}_{3}$$

In 1967, Hausmann first proposed the notion of the chloride corrosion threshold, indicating that when the molar concentration ratio of chloride ions to hydroxide ions (Cl^−^/OH^−^) in the pore solution of concrete surpasses 0.6, the passivation film on the surface of reinforcing steel will deteriorate. This crucial value has now been extensively embraced in engineering practice. This study employs a chloride critical concentration of 0.4% by cement mass fraction [29] as the evaluative criterion.

### 2.1. Electrochemical Processes

#### 2.1.1. Transportation of Substances in Concrete

Assuming the concrete is saturated, the transportation of free chlorides into the interior of the concrete can be calculated using the mass conservation transport equation and adjusted based on the liquid volume percentage. Therefore, the governing equation for the transportation of free chlorides is as follows:

(4) $$\frac{\partial \left(\theta_{l}C_{f}\right)}{\partial t}-\nabla \cdot \left(\theta_{l}D_{f}\cdot \nabla C_{f}+\frac{z_{f}FC_{f}\theta_{l}D_{f}}{RT}\nabla v\right)=\theta_{l}R_{b}\begin{array}{cc} & in\begin{array}{cc} \Omega^{c} & \end{array} \end{array}$$

$D_{f}$ and $z_{f}$ are the diffusion coefficient and charge number of chloride ions, respectively. $\theta_{l}$ is the liquid volume fraction (see Section 2.2.2.). t is time. $C_{f}$ is the chloride ion concentration. F = 96,485 C/mol is Faraday’s constant. R = 8.314 J/(mol K) is the ideal gas constant. T = 298 K is absolute temperature; v is electric potential; free chlorine ions interact with the C-S-H gel to form a complex [37], resulting in an elevation of the concentration of bound chlorine ions, $C_{b}$. The reaction rate of this complexation process is $R_{b}=\langle \alpha (\beta C_{f}-C_{b})\rangle$ [31], $\alpha =10^{-5}[s^{-1}]$ and β=0.7[1] is the Freundlich parameter, while 〈⋅〉 is the Macaulay parameter, as shown in $\langle x\rangle =\frac{x\pm \left|x\right|}{2}$. Bonded chlorides are seen as stationary, and the potential for their liberation from the C-S-H matrix is not considered. Consequently, the concentration of bound chloride ions is determined as

(5) $$\frac{\partial (\theta_{l}c_{b})}{\partial t}=\theta_{l}R_{b}\;\mathrm{in}\;\Omega^{c}$$

Similarly, the migration of multiple ions in saturated concrete can be expressed as

(6) $$\frac{\partial (\theta_{l}C_{i})}{\partial t}=-\nabla \cdot (\theta_{l}J_{i})$$

In the equation, $C_{i}$ and $J_{i}$ are the concentration and flux of ions, respectively; t is time; $\theta_{l}$ is the liquid volume fraction.

The diffusion of various ions in saturated concrete is controlled by two factors: the diffusion resulting from concentration gradients of the ions and the migration induced by external electric fields or electrostatic potentials arising from charge imbalances among the ions. Consequently, the flux of different ions can be articulated by the Nernst–Planck equation:

(7) $$J_{i}=-D_{i}\nabla C_{i}-\frac{z_{i}F}{RT}D_{i}C_{i}\nabla v$$

$D_{i}$ and $z_{i}$ are the diffusion coefficient and charge number of the i-th ion, respectively.

The transport of ionic compounds corresponds to the current in an electrolyte, with current density defined by Faraday’s law.

(8) $$i_{i}=-z_{i}FJ_{i}$$

In an electrolyte environment, the total current density results from the vector superposition of the migratory current densities of individual ions, leading to current equilibrium inside the electrolyte.

(9) $$i=\sum_{i}i_{i}=\frac{F^{2}}{RT}\nabla v\sum_{i}z_{i}^{2}D_{i}c_{i},\;\nabla \cdot i=0$$

A pragmatic method is to regard the electrolyte as a conductor and disregard internal charge transport, so establishing the potential distribution according to Ohm’s law, which adheres to the Laplace equation.

(10) $$\nabla \cdot (\frac{1}{\rho }\nabla v)=0,\;i=-\frac{1}{\rho }\nabla v$$

where the effective resistivity of ρ concrete is considered constant; another representation of the net current density is i=−κ∇v, where κ is the electrolyte conductivity $\kappa =\frac{1}{\rho }$; the solid potential of the electrode is assumed to be $v_{s}$ = 0, and hence the electrode potential is determined to be $E=v_{s}-v=-v$, which allows for the incorporation of electrode polarization.

#### 2.1.2. Electrode Polarization

Corrosion of steel reinforcement leads to metal disintegration at the anode and oxygen absorption at the cathode. According to the Tafel equation, the anode current density $i_{\mathrm{Fe}}$ and cathode current density $i_{O_{2}}$ on the surface of the steel reinforcement can be obtained as follows:

(11) $$i_{\mathrm{Fe}}=i_{\mathrm{Fe}}^{0}\exp\left(2.303\frac{v-v_{\mathrm{Fe}}^{0}}{\beta_{\mathrm{Fe}}}\right)$$

(12) $$i_{O_{2}}=\frac{C_{O_{2}}}{C_{O_{2}}^{s}}i_{O_{2}}^{0}\exp\left(2.303\frac{v_{O_{2}}^{0}-v}{\beta_{O_{2}}}\right)$$

In the equation, $i_{\mathrm{Fe}}$ and $i_{O_{2}}$ (A/m^2^) represent the anode current density and cathode current density, respectively; $i_{\mathrm{Fe}}^{0}$ and $i_{O_{2}}^{0}$ (A/m^2^) denote the anode exchange current density and cathode exchange current density, respectively; $v_{\mathrm{Fe}}^{0}$ and $v_{O_{2}}^{0}$ (V vs. SCE) indicate the equilibrium potential at the anode and cathode, respectively; $\beta_{\mathrm{Fe}}$ and $\beta_{O_{2}}$ (V/dec) represent the Tafel slope at the anode and cathode, respectively. $C_{O_{2}}$ and $C_{O_{2}}^{s}$ (mol/m^3^) represent the dissolved oxygen concentrations on the steel surface and the concrete protective layer surface, respectively. As the chloride ion concentration increases to the threshold value, $\beta_{a}^{\mathrm{Fe}}=\left\{\begin{array}{ll} +\infty , & \mathrm{Passive}\;\mathrm{anode}\\ \beta_{\mathrm{Fe},0}, & \mathrm{Active}\;\mathrm{anode} \end{array}\right.$ can be used to distinguish the active zone and the passivation zone on the steel surface [38]. An infinite value is represented as 50 V/dec [28].

#### 2.1.3. Electrochemical Corrosion of Reinforcing Steel

Assuming that the concrete is saturated, it is described by representative volume elements (RVEs), and the concrete porosity $p_{0}=(V-V_{s})/V$ is considered constant. $V_{s}$ is the volume of the concrete RVE and is divided into liquid pore solution and gradually accumulating solid iron precipitates (rust). The liquid pore solution is $\theta_{l}=V_{l}/V$ and the gradually accumulated solid iron precipitate (rust) content is $\theta_{p}=V_{p}/V$.

The distribution of Fe^2+^ and Fe^3+^ ions in concrete is represented by $C_{II}$ and $C_{III}$, respectively, ignoring the small deformation velocity of the concrete matrix. The governing equations describing the mass transport models of these two substances are as follows:

(13) $$\left(\theta_{l}D_{X}\cdot \nabla C_{X}+\frac{z_{X}FC_{X}\theta_{l}D_{X}}{RT}\nabla v\right)=\theta_{l}R_{X}\begin{array}{cc} & \begin{array}{cc} in\Omega^{c} & X=II,III \end{array} \end{array}$$

$D_{X}$ and $z_{X}$ are the diffusion coefficient and charge number of the xth ion, respectively.

The newly produced precipitate AP gradually occupies the pores, diminishing the pore space accessible for diffusion and chemical reactions. The precipitate is deemed stationary, and its progression adheres to the subsequent equation:

(14) $$\frac{\partial \theta_{p}}{\partial t}=\frac{M_{p}}{\rho_{p}}\theta_{l}R_{p}\;\mathrm{in}\;\Omega^{c},\;R_{p}=K_{r}^{III\to p}C_{III}$$

where $M_{p}$ and $\rho_{p}$ are the molar mass and density of the precipitate, respectively; the initial values of $C_{II}$, $C_{III}$, and $\theta_{P}$ are assumed to be zero.

On the corrosively active steel boundary *Γ*^*s*^, the inflow of ferrous ions is calculated according to Faraday’s law as follows:

(15) $$J_{II}=\frac{i_{a}}{z_{a}F}=n\cdot D_{II}\cdot \nabla C_{II}$$

where $i_{a}$ is the corrosion current density. $z_{a}$ = 2 indicates the number of electrons exchanged by each iron atom in the anodic reaction; on the remaining boundaries, the flux of $C_{II}$ and $C_{III}$ is zero.

The reaction rate of a chemical reaction follows the following law: $R=k_{r}\prod_{i=1}^{N}C_{i}^{X_{i}}$, where $k_{r}$ is the rate constant, $C_{i}$ is the concentration of the reactant, and $X_{i}$ is the exponent determined experimentally. The reaction rate of the continuous conversion from ferrous ions to precipitates [30] can be described as

(16) $$R_{II}=-K_{r}^{II\to III}C_{II}C_{o^{2}}$$

(17) $$R_{III}=-R_{II}-R_{p}=K_{r}^{II\to III}C_{II}C_{o^{2}}-K_{r}^{III\to p}C_{III}$$

### 2.2. Fracture Phase Field Model

#### 2.2.1. Unified Phase Field Theory

The unified phase field model (PF-CZM) approach is utilized to simulate chloride-induced cracking in reinforced concrete, incorporating the non-homogeneity of the material. This method successfully combines electrochemical reactions with mechanical processes, allowing a thorough and cohesive modeling of corrosion-induced cracking. In this study, the damage evolution of the concrete matrix and the interface transition zone (ITZ) is described using the phase field regularized cohesive zone model proposed by Wu [25]. Aggregates and reinforcement are presumed to adhere to linear elastic constitutive relations, disregarding their plastic deformation and damage progression, to concentrate on the failure mechanism analysis of the interfacial transition zone and matrix materials. By disregarding body forces, the total energy throughout the domain and on the fracture surface can be articulated as

(18) $$\prod \left(u,d\right)=\int_{\Omega }\psi (\varepsilon (u),d)d\Omega +G_{f}\int_{\Omega }\gamma (d,\nabla d)d\Omega -\int_{\partial \Omega_{\epsilon }}t\cdot \mathrm{ud}\partial \Omega_{\epsilon }$$

Among these, $G_{f}$ represents the material fracture energy; d denotes the phase field scale, and indicates the damage level, with d = 0 representing no damage and d = 1 indicating complete damage; γ(d,∇d) is the crack surface density function; $\partial \Omega_{\epsilon }$ denotes the boundary of the domain Ω; t is the surface force vector on $\partial \Omega_{\epsilon }$. u is the displacement field; ε is the strain tensor; and ψ(ε(u),d) is the modified energy density function.

According to the principle of minimum energy, the following function can be obtained from the variation of Equation (18):

(19) $$\begin{array}{c} \delta \prod (u,d)=\int_{\partial \Omega_{t}}(\sigma (u,d)n-t)\delta ud\partial \Omega_{t}-\int_{\Omega }\mathrm{div}\sigma (u,d)\delta ud\Omega \\ \begin{array}{l} +\int_{\Omega }[-G_{f}\nabla \cdot (\frac{\partial \gamma (d,\nabla d)}{\partial \nabla d})+G_{f}\frac{\partial \gamma (d,\nabla d)}{\partial d}+\frac{\partial \psi (\varepsilon (u),d)}{\partial d}]\delta dd\Omega \\ +\int_{\partial \Omega }G_{f}\frac{\partial \gamma (d,\nabla d)}{\partial \nabla d}n\delta dd\partial \Omega \end{array} \end{array}$$

The displacement field u in the above equation should satisfy the displacement boundary conditions in advance. Since the displacement field u and the phase field d are two independent variables, the basic equations and boundary conditions that the displacement field and phase field should satisfy can be given.

The displacement field equation is

(20) $$\nabla \cdot \left[\omega \left(d\right)\sigma \right]+f=0\begin{array}{cc} & in \end{array}\Omega$$

(21) $$\omega \left(d\right)\sigma \cdot n=\bar{t}\begin{array}{cc} & in \end{array}\partial \Omega_{t}$$

(22) $$u=\bar{u}\begin{array}{cc} & in \end{array}\partial \Omega$$

The phase equation is

(23) $$\omega^{\prime }(d)\psi (\varepsilon )+\frac{G_{f}}{l_{0}}(d-l_{0}^{2}\Delta d)=0\begin{array}{cc} & in \end{array}\Omega$$

(24) $$\nabla d\cdot n=0\begin{array}{cc} & in \end{array}\partial \Omega$$

Among these, $\omega \left(d\right)$ is the phase field degradation function; Δ is the Laplace operator; $\partial \Omega_{u}$ is the boundary for the given displacement; $\partial \Omega_{t}$ is the boundary where the external load is applied; the boundary of the entire solution domain Ω is $\partial \Omega =\partial \Omega_{u}+\partial \Omega_{t}\left(\partial \Omega_{u}\cap \partial \Omega_{t}=d\right)$; $l_{0}$ is the phase field characteristic width; n is the unit normal vector of the boundary, while $\bar{u}$ and $\bar{t}$ are the known displacement or external force values on the corresponding boundaries, respectively. Let

(25) $$F_{d}=-\omega^{\prime }(d)\psi (\varepsilon )$$

Known as the phase field evolution driving force, when it is unloaded, the phase field value may decrease, causing cracks to heal themselves, which is clearly unreasonable. Therefore, a historical variable is introduced to replace the energy density function in Equation (23).

The degeneracy function used in the phase field model must satisfy the following conditions: 1. $\omega \left(0\right)=1$ represents regions where the phase field value is 0, indicating that the material has no damage, i.e., the strain potential energy has not been reduced; 2. $\omega \left(1\right)=0$ represents regions where the phase field value is 1, indicating that the material is completely damaged and no longer has any load-bearing capacity; i.e., the strain potential energy should be 0; 3. $\omega^{\prime }(d)=0$ represents regions where the phase field value cannot evolve further after complete material failure; 4. $\omega^{\prime }(d)<0$ represents regions where material damage increases with increasing phase field values, and the reduction coefficient of strain energy should decrease accordingly. Therefore, the degradation function should be a strictly monotonically decreasing function of the phase field value. This essay adopts the degradation function used in the unified phase field model [26] to describe the energy degradation process:

(26) $$\omega \left(d\right)=\frac{{\left(1-d\right)}^{p}}{{\left(1-d\right)}^{p}+Q\left(d\right)}$$

Among them, index p>0 is a constant to be determined, while continuous $Q\left(d\right)>0$ takes the form of

(27) $$Q(d)=a_{1}d+a_{1}a_{2}d^{2}+a_{1}a_{2}a_{3}d^{3}=a_{1}dP(d)$$

For the phase field intensity function, its definition is as follows:

(28) $$\gamma (d,\nabla d)=\frac{1}{c_{0}}\left[\frac{\alpha (d)}{l_{0}}+l_{0}|\nabla d{|}^{2}\right]$$

Among them

(29) $$c_{0}=4\int_{0}^{1}\sqrt{\alpha \left(s\right)}ds$$

(30) $$\alpha (d)=\xi d+(1-\xi )d^{2}$$

$a_{i}(i=1,2,3)$ is a constant related to material parameters and the softening relationship used. In this essay, the Cornelissen-type cohesive softening relationship is adopted. The Cornelissen-type cohesive softening curve was calibrated by Cornelissen et al. based on concrete experimental data and is therefore widely used in concrete structures. In the Cornelissen-type cohesive softening relationship, p=2, $a_{2}=1.3868$, $a_{3}=0.6567$, ξ=2.

After modifying the degradation function and the surface density function, the phase field evolution Equation (23) should be modified to

(31) $$\frac{G_{c}}{c_{0}l_{0}}\left[\alpha^{\prime }\left(d\right)-2l_{0}\Delta d\right]=-\omega^{\prime }\left(d\right)\psi \left(\varepsilon \right),\begin{array}{cc} & in \end{array}\;\Omega$$

#### 2.2.2. Constitutive Relationship Based on Characteristic Strain

Within the elastic range, the macroscopic stress far from the interface, induced by the evolution of the precipitate phase, can be incorporated into its eigenstrain $\varepsilon_{s}$. At this time, the total strain ϵ is the sum of the elastic strain $\varepsilon_{e}$ and the precipitate-induced strain $\varepsilon_{s}$, that is, $\epsilon =\varepsilon_{e}+\varepsilon_{s}$.

(32) $$\sigma =\omega (d)E_{0}:\epsilon_{e}=\omega (d)E_{0}:\left(\epsilon -\varepsilon_{s}\right)$$

There is a linear dependence relationship between the inelastic strain $\varepsilon_{s}$ induced by the precipitate and the precipitate saturation $S_{p}$:

(33) $$\varepsilon_{s}=\epsilon_{s}1=f\left(S_{p},\phi ,\ldots \right)1\approx CS_{p}1$$

The complicated eigenstrain function $\left(f(S_{p},\phi ,\ldots \right)$ can be simplified to a positive scalar constant C, and it is assumed that C is proportional to the volumetric strain $\epsilon_{V}$.

(34) $$\epsilon_{V}=\frac{V_{p}}{(1-p_{p})V_{\mathrm{III}}}-1=\frac{\rho_{\mathrm{III}}M_{p}}{(1-p_{p})\rho_{p}M_{\mathrm{III}}}-1$$

Herein, the molar volume $V_{p}=M_{p}/\rho_{p}$, and $p_{p}$ is the porosity of the precipitate.

Assuming that the concrete is isotropic, the specific eigenstrain $\varepsilon_{p,0}$ can be defined as

(35) $$\epsilon_{p,0}=\frac{1}{3}\epsilon_{V}S_{p}1$$

To distinguish the material properties of rust from those of concrete, for the regions not affected by the boundary surface traction $t^{*}$, the eigenstrain is [29]

(36) $$\varepsilon_{s}=\frac{3(1-\nu )K_{p}}{(1+\nu )K_{p}+(2-4\nu )K_{c}}\varepsilon_{p,0}$$

Therefore, the eigenstrain $\varepsilon_{s}$ of the precipitate phase can be expressed as

(37) $$\varepsilon_{S}=CS_{p}1,\mathrm{with}\;C=\frac{(1-\nu )K_{p}}{(1+\nu )K_{p}+(2-4\nu )K}\left(\frac{\rho_{III}M_{p}}{(1-r_{0})\rho_{p}M_{III}}-1\right)$$

where $r_{0}$ is the expansion rate of rust; the bulk modulus $K_{p}=\frac{E_{p}}{3(1-2\nu_{p})}$ of the precipitate is the bulk modulus of the rust precipitate calculated from the Young’s modulus $E_{p}$ and Poisson’s ratio $\nu_{p}$ of the rust, and similarly, the bulk modulus $K=\frac{E}{3(1-2\nu )}$ of the rust product-filled concrete is calculated from the Young’s modulus E and Poisson’s ratio ν of the rusted concrete. Since the mechanical properties of rusted and rust-free concrete are very different, the Young’s modulus E and Poisson’s ratio ν of the rust-filled concrete are given by

(38) $$E=(1-\theta_{p})E_{c}+\theta_{p}E_{p},\;\nu =(1-\theta_{p})\nu_{c}+\theta_{p}\nu_{p}$$

For a concrete cube specimen with built-in reinforcement, the crack opening width w can be estimated by integrating the inelastic strain $E_{d}$ over the concrete surface $\Gamma_{C}$ [39], using the upper concrete surface $\Gamma_{C}^{u}$ as an example.

(39) $$w=\int_{\Gamma_{C}^{u}}{\left(\varepsilon_{d}\right)}_{x}d\Gamma =\int_{\Gamma_{C}^{u}}\left(1-\omega (d)\right)\left(\varepsilon_{x}-{\left(\varepsilon_{d}\right)}_{x}\right)d\Gamma$$

$\varepsilon_{d}=\varepsilon -\varepsilon_{e}-\varepsilon_{s}$ in the formula.

#### 2.2.3. Crack-Induced Diffusion Coefficient Variation

The cracks provide a convenient channel for the transport of a wide range of ions, thus increasing the diffusion coefficients of the various ions. This relationship can be expressed as

(40) $$\theta_{l}D_{\alpha }=\theta_{l}(1-d^{n})D_{m,\alpha }1+d^{n}D_{c,\alpha }1$$

α represents various ions, where $D_{m,\alpha }$ is the diffusion coefficient of the considered substance in concrete and $D_{c,\alpha }$ is the diffusion coefficient of the parametrically controlled cracking material; where $D_{c,\alpha }$ is much larger than $D_{m,\alpha }$, assuming that diffusion coefficients of the mediums in the transition zone and in the cracks are three times larger than those of the mediums in the matrix [40,41] and 10 times larger [42], and that the chloride ion n is taken to be 15, and the rest of n is taken to be 1 [43].

## 3. Numerical Validation

### 3.1. Numerical Implementation

The numerical implementation was conducted following the development of the theoretical framework to evaluate the model’s accuracy and predictive capability. This study analyzes concrete as a multi-phase structure comprising mortar, aggregate, and transition zones. The experiments utilized a continuous gradation ranging from 150 to 1180 μm to produce various aggregate levels, with particles smaller than 150 μm classified as fine aggregates for incorporation into the mortar. The concrete aggregates were generated through random aggregate distributions based on modified Fuller grading curves in MATLAB R2024. The distribution was eventually exported to COMSOL Multi-physics 6.3 for numerical simulations. Diffusion and electrochemical processes were executed through a cubic current distribution, known as the Nernst–Planck module. The dilute substance transport module conducted continuous chemical operations. The mechanical and cracking processes were defined by the solid mechanics and Poisson equation modules, respectively. An interleaving algorithm was applied at each incremental time step. Initially, the concentrations of oxygen and chloride ions were ascertained, followed by the calculation of electrolyte potential and corrosion current density. Subsequently, the intrinsic relationship was established via the strain field to enable the fracture phase field equations to precisely characterize the crack propagation process.

**Figure 2:**
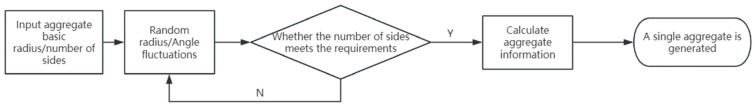
Random concave-convex aggregate generation process.

In order to truly reflect the fine structural characteristics of concrete, this study uses random convex-concave polygons to simulate aggregate particles, defining the position of any vertex $P_{i}$ in the two-dimensional plane using polar coordinates $\left(r_{i},\theta_{i}\right)$ (refer to Equation (41) for the vertex coordinate generation method), which are subsequently transformed into Cartesian coordinates through coordinate transformation. For polygons with more than two vertices (n > 2), the total area of the polygon aggregate is computed using the triangular area accumulation method, with the detailed generation process illustrated in Figure 2.

(41) $$\left\{\begin{array}{c} r:r_{i}\times \left[\begin{array}{c} 1+\mathrm{random}(-1,1)\times f_{r} \end{array}\right]\\ \theta :\frac{2\pi }{\alpha }\times \left[\begin{array}{c} \beta +\mathrm{random}(-1,1)\times f_{\theta } \end{array}\right] \end{array}\right.$$

r is the random radius of any point $P_{i}$, $f_{r}$ is the radius fluctuation ratio; $f_{\theta }$ is the angle fluctuation ratio, α is the number of aggregate edges, β is the number of aggregate corner points, and random() is a randomized function indicating the generation of a random number between (−1,1).

Fuller’s curve is acknowledged as the optimal grading curve for concrete; however, it is applicable solely to three-dimensional scenarios. This study focuses on two-dimensional planes, employing Walraven’s [44] Equation (42) to transform Fuller’s gradation into a two-dimensional particle size grading curve, which is presented as follows.

(42) $$\begin{array}{l} P(D<D_{0})=P_{k}[1.065{\left(\frac{D_{0}}{D_{\max}}\right)}^{0.5}-0.053{\left(\frac{D_{0}}{D_{\max}}\right)}^{4}\\ -0.012{\left(\frac{D_{0}}{D_{\max}}\right)}^{6}-0.0045{\left(\frac{D_{0}}{D_{\max}}\right)}^{8}+0.0025{\left(\frac{D_{0}}{D_{\max}}\right)}^{10}] \end{array}$$

where $P(D<D_{0})$ is the percentage of aggregate area that passes through the sieve diameter $D_{0}$, $P_{k}$ is the percentage of aggregate area to the total area, and $D_{\max}$ is the maximum aggregate particle diameter.

### 3.2. Verification of Corrosion-Induced Cracking of the Protective Layer of Concrete

**Figure 3:**
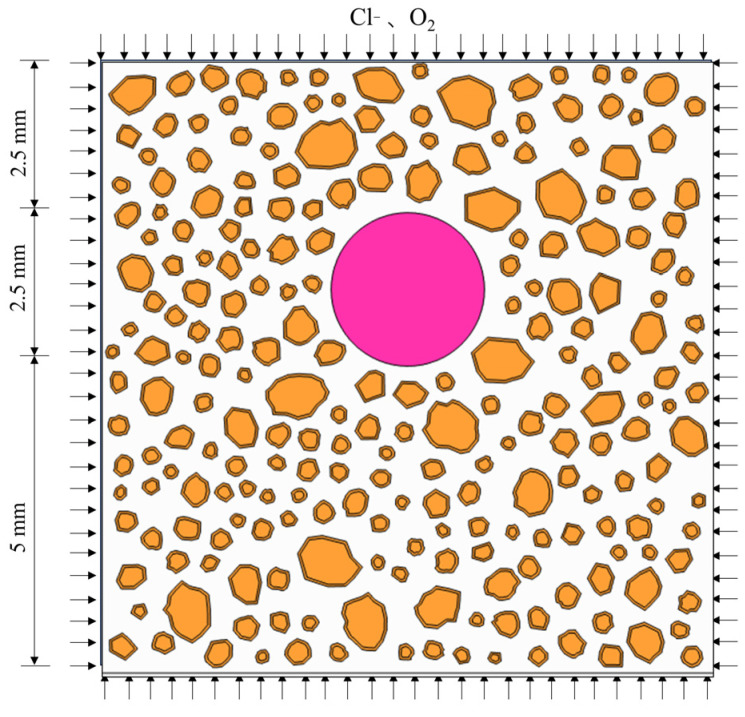
Geometry and boundary conditions of phase field model.

This study employs the experimental results from [45] to computationally simulate and validate the corrosion of steel bars and the cracking of concrete surfaces induced by chloride ions. The experiment initially submerged the samples in a 3.5 wt% NaCl solution, alternating between wetting and drying cycles to expedite the corrosion process. It utilized XCT to obtain corrosion slices at 72-hour intervals. The chloride ion and oxygen concentrations were consistently established at 2 wt% and 0.268 mol/m^3^ [29] on the surface of concrete examples, with the bottom surface fixedly constrained. The simulation evaluated the transport of six ions in concrete: Cl^−^, Na^+^, Ca^2+^, OH^−^, O_2_, and K^+^. The numerical model’s geometric shape and boundary conditions are shown in Figure 3. White represents mortar, orange represents aggregate and transition zone, and pink represents steel reinforcement.

The surfaces of the concrete specimens are exposed to a chloride environment, and the displacements in both the vertical and horizontal axes at the bottom surface are constrained. The parameters used for phase field, electrochemistry, and diffusion are shown in Table 1, Table 2 and Table 3. The phase field characteristic width is 250 µm [29], and the ITZ thickness is 50 µm [46].

**Table 1:** Electrochemical parameters in the simulation [15,47].

Parameters	Value
Anodic Tafel slop, $\beta_{Fe}\left(V/dec\right)$	0.09
Cathodic Tafel slope, $\beta_{O_{2}}\left(V/\mathrm{dec}\right)$	−0.14
Anodic equilibrium potential, $\Phi_{Fe}\left(V\right)$	−0.78
Cathodic equilibrium potential,${\;\Phi }_{O_{2}}\left(V\right)$	0.16
Anodic exchange current density, $i_{0,\mathrm{Fe}}\left(A/m^{2}\right)$	3 × 10^−4^
Cathodic exchange current density, $i_{0,O_{2}}\left(A/m^{2}\right)$	1 × 10^−5^
Concrete resistivity, $\rho_{c}\left(\Omega m\right)$	200

**Table 2:** Diffusion parameters in simulation [39].

Variables	Cl^−^	Na^+^	Ca^2+^	OH^−^	K^+^	O_2_
Charge number	−1	1	2	−1	1	-
Diffusion coefficient (×10^−11^ m^2^/s)	1.2	1.33	0.79	5.27	1.96	600
Initial concentration (mol/m^3^)	0	40	15	140	70	0.156
Boundary concentration (mol/m^3^)	-	-	0	0	0	0.268

**Table 3:** Phase field parameters [48,49].

Phases	Young’s Modulus (MPa)	Poisson’s Ratio	Failure Strength (MPa)	Fracture Energy (N/m)	Porosity
Aggregate	70,000	0.2	-	-	-
Matrix	25,000	0.2	3	40	0.26
ITZ	15,000	0.2	1.5	20	0.26
Precipitate	440	0.4	-	-	0.16

**Figure 4:**
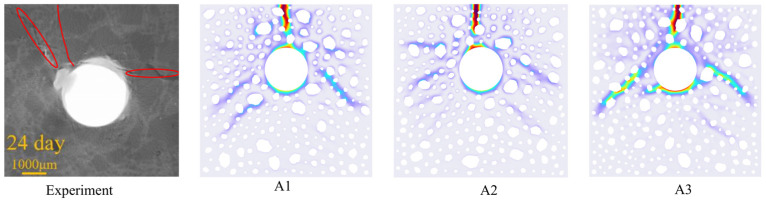
Simulation results and experimental comparisons of different aggregate distributions.

A comparative analysis of the crack morphology obtained from experimental observations and numerical simulations is illustrated in Figure 4. This effectively validates the model’s reliability, as the crack morphology derived from numerical simulation is in excellent agreement with the experimental results. The numerical model depicts the crack extension paths in the lower left and lower correct orientations, which were not observed in the experiments. The curvature of the crack extension paths is influenced by the spatial distribution characteristics of the aggregate, which alters the distribution of the stress field. The aggregate, a high-stiffness phase, significantly increases the complexity of crack extension paths. However, its impact on the overall direction of crack development is relatively limited.

**Figure 5:**
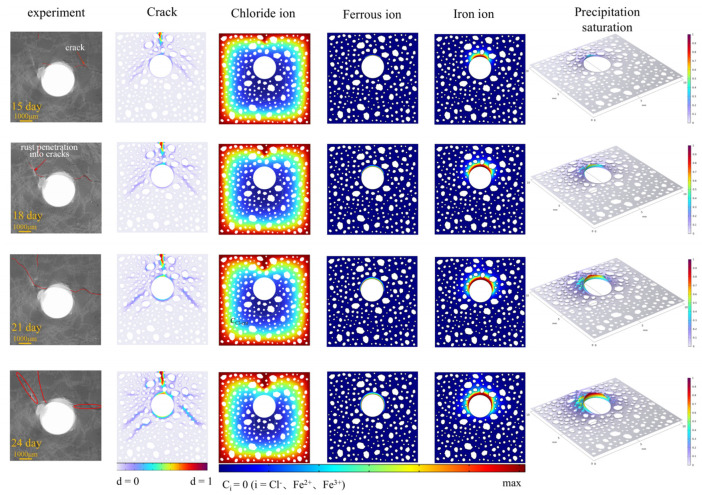
The various stages in experiments and simulations.

Figure 5 illustrates the stage-like characteristics of corrosion progression in reinforced concrete, as evidenced by experimental observations and numerical simulations. This is achieved through a comparative analysis of multidimensional parameters, including the distribution of rust products, crack propagation patterns, chloride concentrations, and the intricate coupling behavior of Fe^2+^/Fe^3+^ ion concentration fields. The multitude of physical field components form a standard multi-physical field interaction system, illustrating the complicated mechanisms of electrochemical–mechanical coupling processes inside the model. Research indicates that when the chloride concentration in concrete pore solutions attains the critical threshold CCI-crit = 0.4 wt% (shown by the white line in the figure), it triggers chloride-induced corrosion of reinforcement. This threshold has substantial spatiotemporal relationships with the initial stages of corrosion and the progression of cracks. At the critical times of 15 and 24 days, the fracture propagation patterns observed from the experiment and simulation exhibited remarkable congruence. After 21 days, the crack morphology exhibited a notable transformation: transitioning from the initial focused propagation of two primary cracks to a diffuse damage distribution optimal for energy dissipation. After 21 days, the crack morphology exhibited a notable transformation: transitioning from the initial focused propagation of two primary cracks to a diffuse damage distribution optimal for energy dissipation. When the energy necessary to propagate existing cracks surpasses the threshold for initiating new cracks, the system typically dissipates strain energy as diffuse damage, resulting in a “divergent” cracking pattern that contrasts with experimental findings. The level of damage influences the transit of Fe^2+^ ions; however, the ongoing oxidation process converting Fe^2+^ to Fe^3+^ diminishes the direct relationship between the concentration distribution of this ion and the damaged area to a certain degree.

**Figure 6:**
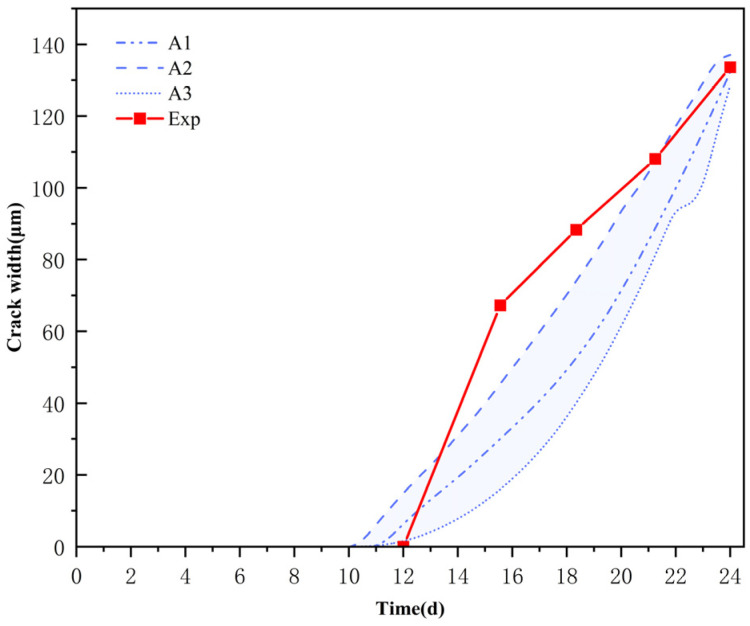
The influence of different distributions of aggregates on concrete cracking penetration, ultimately resulting in a strong convergence between experimental and simulated data.

Figure 6 compares the development of concrete surface crack width across various aggregate distributions and corresponding experimental outcomes. In the initial 18 days of the corrosion process, the numerical results were predominantly lower than the experimental findings. Beginning on the 18th day, the gap between the two progressively diminished, and by the 24th day, the relative error between the calculated crack width and the experimental measurement was maintained within 5%, with both exhibiting a very consistent pattern in fracture propagation. This suggests that the approach indicated in this work may successfully capture the dynamic growth patterns of corrosion fractures, exhibiting strong applicability and dependability in long-term predictions. The crack development curves of the three aggregate distribution models (A1, A2, and A3) demonstrate consistency throughout the overall progression, with the final crack width variations remaining within a 3% range. This shows that the spatial arrangement of aggregates has little influence on the ultimate cracking width. The comparison of the initial cracking timings indicates that the numerical model forecasted an initial cracking time of 11.5 days, closely aligning with the 12 days recorded in the experiment, resulting in an error rate of merely 4.15%. In the first 20 days of the simulation, the numerical calculation results were slightly lower than the experimentally measured values. This concerns the model’s inadequate consideration of the bridging effect of early hydration products on fissures. As the time reached beyond 18 days, the disparity between the two diminished progressively due to the accumulation of corrosion products and the escalation of cracks. There is an influence of different distributions of aggregates on concrete cracking penetration, ultimately resulting in a strong convergence between experimental and simulated data at 24 days. The established numerical model can effectively capture the crack initiation characteristics and long-term evolution trends under the influence of aggregate distribution, providing a reliable method for the micro-scale simulation of corrosion cracking in concrete structures.

Based on the electrochemical–mechanical coupling model framework proposed by Korec [31], numerical methods comparable with the original literature were employed to simulate the comparative experiment model. During the simulation, the original parameter settings were rigorously followed: the diffusion coefficient and phase field damage parameters were completely consistent with the model in this study, where T was set to 0.56%, and the anode current density ia was 4.6 μA/cm^2^.

**Figure 7:**
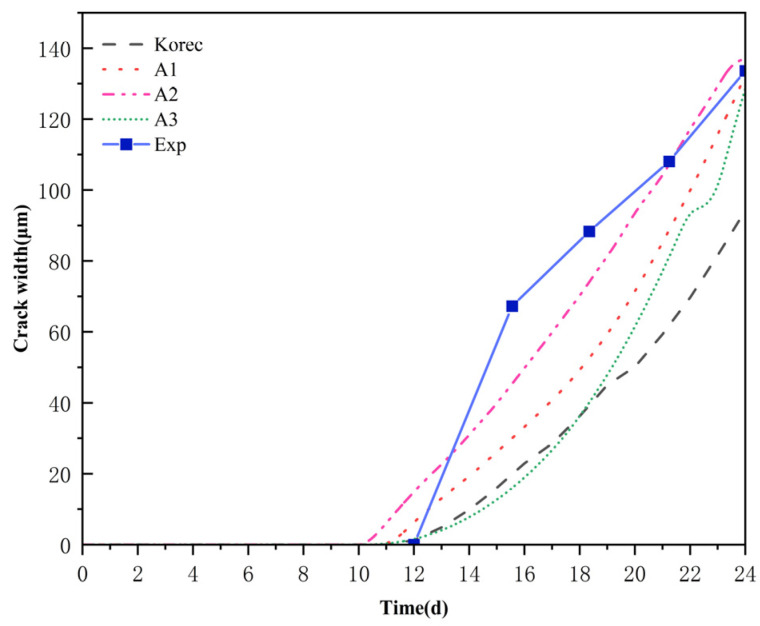
Comparison chart of crack widths in Korece [31]’s models.

Figure 7 illustrates that the crack width evolution curve predicts the initial cracking time according to the Korec model to be 11.5 days, which aligns closely with the experimental results of 12 days, yielding a relative error of merely 4.2%, thereby validating the model’s efficacy in representing the corrosion-induced cracking phase. During the crack propagation phase, the Korec model predicted a maximum crack width of 94 μm, which markedly deviated from the experimentally measured maximum value of 133 μm, resulting in a relative error of 29%. This deviation was primarily the result of the original model failing to account for the damage-dependent diffusion coefficient in the interface transition zone (ITZ), the deflection effect of aggregate distribution on fracture paths, and the variable corrosion current density. In contrast, the experimental data is consistent with the prediction results of the enhanced model in this essay, with a maximal crack width error of only 5%. The remarkable decrease in error magnitude unequivocally demonstrates the efficacy and relevance of the enhancements presented in this research, underscoring the model’s capacity to effectively replicate the cracking process of concrete in intricate corrosion conditions.

### 3.3. Ion Concentration Distribution Verification

**Figure 8:**
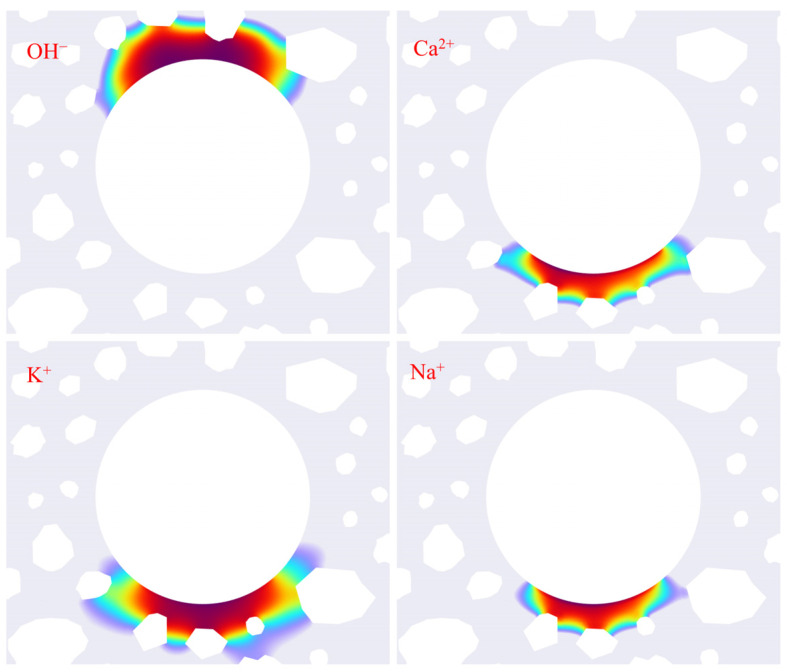
Ion concentration distribution diagram.

Figure 8 illustrates the distribution features of ion concentrations in the vicinity of the reinforcing bars under various aggregate distribution models A1 on the 24th day. Upon meticulous research of this map, it is evident that hydroxide ions (OH^−^) display pronounced spatial aggregation, predominantly concentrated near the concrete protective layer. In contrast, cations, specifically calcium ions (Ca^2+^), are predominantly located on the side distal to the protective layer. This distribution pattern aligns well with the findings of Liu Qingfeng [50]. From an electrochemical mechanism point of view, within the steel–concrete interface system, the reduction reaction of the galvanic cell transpires on the side adjacent to the protective layer, functioning as the positive electrode of the galvanic cell. By the principles of electrochemical processes, anions proceed toward the positive electrode. Conversely, on the side distal to the protective layer, functioning as the negative electrode, an oxidation reaction transpires, propelling cations into that area. The experimental findings on ion distribution align closely with electrochemical theoretical predictions, offering substantial support for the model developed in this study.

## 4. Results and Discussion

### 4.1. Mechanism of ITZ Strength on Crack Expansion Mode of Reinforced Concrete

**Figure 9:**
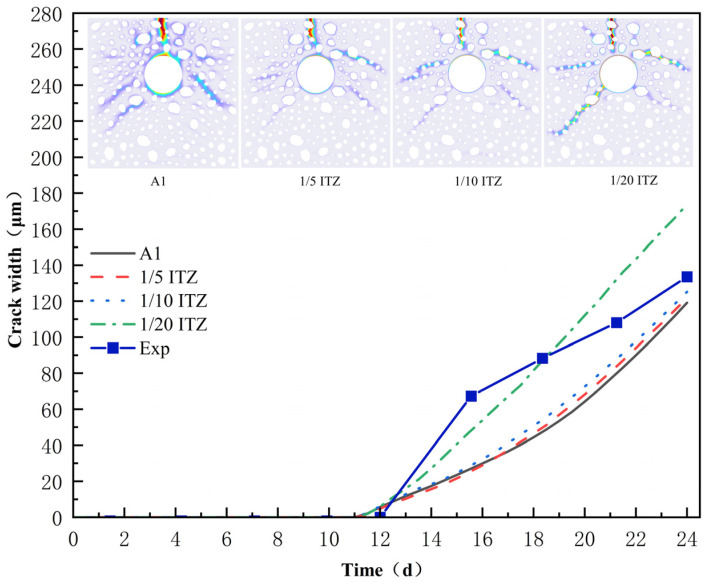
The influence of ITZ.

The interfacial transition zone (ITZ) in the concrete fine structure system works as a crucial bond between the aggregate and the cementitious matrix, with its mechanical properties, including tensile strength and fracture energy, being substantially influenced by factors such as the mineral composition of the aggregate and surface roughness [51,52,53,54,55]. As shown in Figure 9, when the ITZ’s tensile strength and fracture energy are established at 1/5, 1/10, and 1/20 of the mortar matrix, respectively, all other parameters remain consistent with A1. The initially observed cracking patterns exhibited minor changes, and the influence of ITZ strength factors on the crack initiation time was insignificant. Nonetheless, as the tensile strength and fracture energy of the interfacial transition zone (ITZ) diminished, the primary crack propagation path exhibited a pronounced aggregate bias. When the ITZ strength was reduced to 1/10 of that of the mortar matrix, the main crack began to extend near the aggregate boundary; further reduction to 1/20 of the ITZ strength resulted in the crack traversing the aggregate. This behavior results from the stress redistribution effect due to the strength gradient between the interfacial transition zone (ITZ) and the mortar matrix, wherein the low-strength ITZ fails to adequately transfer interfacial stress, leading to the relocation of the stress-concentrated region at the crack tip into the aggregate’s interior. It is noteworthy that when the ITZ strength parameter is 1/20, a left-sloping downward secondary fracture emerges in the crack pattern, indicating the simultaneous expansion of several cracks caused by ITZ failure.

The evolution of crack width (Figure 9) indicates that while the interfacial transition zone (ITZ) strength is 1/5 or 1/10 of the mortar matrix, the maximum crack width remains relatively constant. However, as the strength ratio diminishes to 1/20, there is a pronounced increase in the maximum fracture width. This phenomenon validates the need for fine-scale simulation, as the mechanical properties of the interfacial transition zone (ITZ) not only substantially influence the selection of crack propagation paths but also serve as a critical factor in regulating abrupt variations in crack width under extreme low-strength conditions, thereby offering a significant theoretical foundation for the optimization and management of ITZ in concrete durability design.

### 4.2. Effect of Oxygen Diffusion Coefficient on Corrosion Cracking

**Figure 10:**
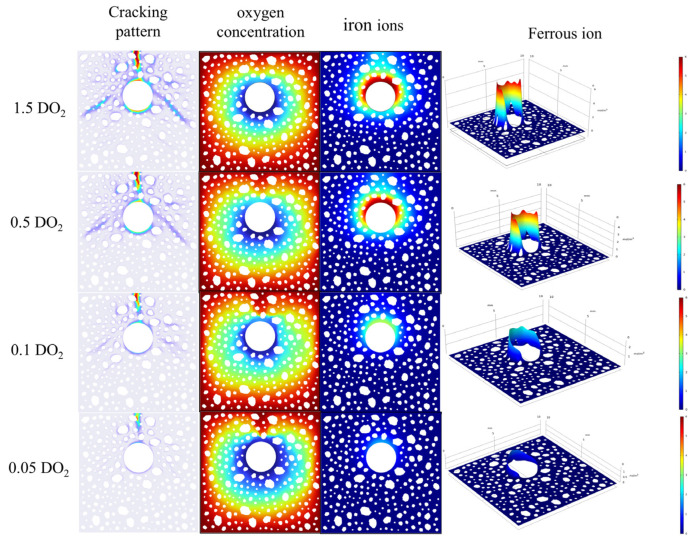
The influence of different oxygen diffusion rates.

The evolution of the cracking pattern at day 24 under different oxygen diffusion coefficients is shown in Figure 10. The cracking patterns exhibited a significant degree of resemblance on the macroscopic scale when the oxygen diffusion coefficients were 0.5 DO_2_ and 1.5 DO_2_, respectively. This indicates that at elevated oxygen diffusion capacity, the cracking process is not much limited by oxygen transport situations, and its progression is mostly governed by other non-oxygen diffusion parameters. Conversely, when the oxygen diffusion coefficient is diminished to 0.05 DO_2_ and 0.1 DO_2_, the extent of damage to the concrete structure exhibits a notable reduction. Particularly at an oxygen diffusion rate of 0.05 DO_2_, only a singular primary crack manifests on the surface of the structure. This occurrence can be ascribed to the alteration in the driving force mechanism of crack propagation; under conditions of diminished oxygen diffusion, crack expansion is predominantly governed by mechanical stresses induced by the expansion of corrosion products.

**Figure 11:**
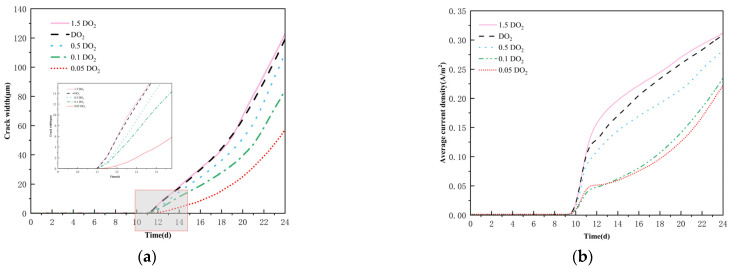
(**a**) Crack width under different oxygen diffusivity; (**b**) The average current density under different oxygen diffusion rates.

In conditions of low oxygen diffusion, crack propagation is primarily governed by the mechanical stress resulting from the expansion of corrosion products. Although oxygen diffusion is constrained, the localized region can sustain a continuous corrosion reaction, leading to the formation of a singular primary crack, as the generated expansion stress is insufficient to trigger the concurrent development of multiple cracks. Meanwhile, analyzed in combination with the crack width evolution curves in Figure 11a, it is evident that while varying oxygen diffusion coefficients hardly influence the initial cracking time, they markedly affect the end crack width. A decrease in the oxygen diffusion coefficient obstructs oxygen transport, resulting in a decreased cathodic reaction rate, which subsequently reduces the average corrosion density of the reinforcement bars. This conclusion is corroborated by the comparative curves of average corrosion densities at varying oxygen diffusion rates in Figure 11b. The diminished creation of corrosion products results in an inadequate driving force for crack propagation, ultimately leading to a reduction in both the extent of damage and the width of cracks in the concrete structure.

### 4.3. Effect of Protective Layer Thickness and Reinforcement Diameter on Corrosion Cracking

**Figure 12:**
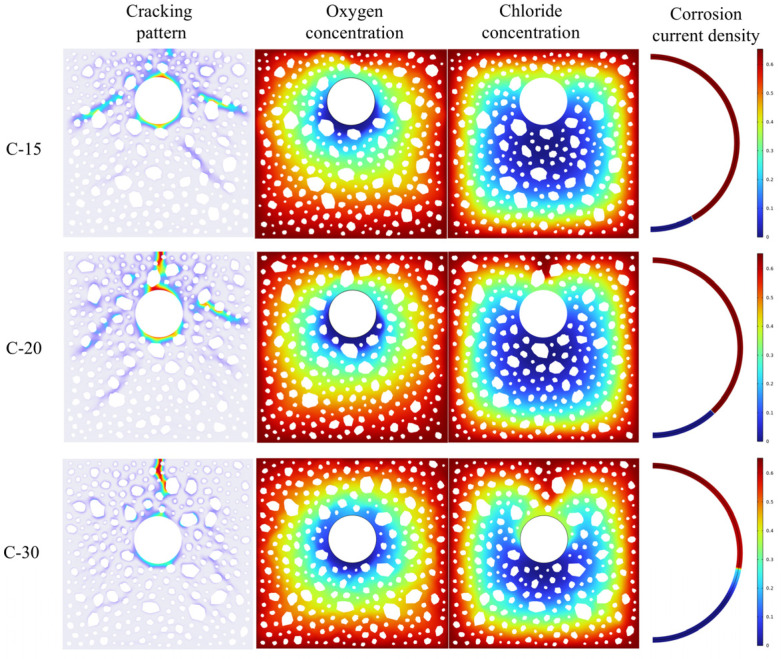
The influence of different protective layer thicknesses.

Figure 12 shows the crack development pattern and current density distribution of reinforced concrete on day 24 under concrete protective layer thicknesses of 1.5 mm (C-15), 2 mm (C-20), and 3 mm (C-30), and a reinforcement diameter of 2.5 mm. The larger protective layer (C-30) results in a prolonged arrival time of the chloride ions, which results in a delayed effect. Additionally, the activation area of the reinforcement is smaller than that of C-20 and C-15, resulting in only one upward primary crack. C-15 demonstrates the exact opposite behavior due to its shortened transport distance. The anomalous fracture pattern observed in C-15 may result from the coupled effect of reduced cover thickness and subsequent changes in aggregate distribution.

**Figure 13:**
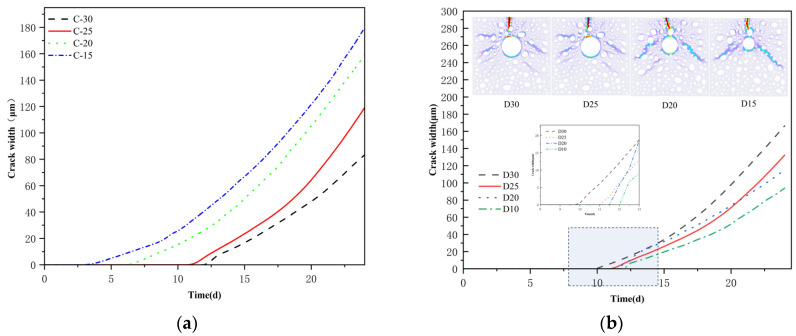
(**a**) Crack width curves with different protective layer thicknesses; (**b**) Crack width under different steel bar diameters.

The thickness of the concrete protective layer exhibited a substantial nonlinear relationship with the surface crack width, as illustrated in Figure 13a, which was influenced by the delayed effect of chloride ion erosion. The crack initiation time is substantially advanced, and the crack width growth rate exponentially increases as the protective layer thickness decreases. This phenomenon suggests that the reduction in the thickness of the protective layer not only expedites the process of chloride erosion but also exacerbates the accumulation of rust and expansion stresses. Consequently, the concrete structure deteriorates at a rate significantly faster than the linear law predicted.

The effect of the reinforcing diameter on the cracking behavior of concrete was methodically investigated for diameters of 3 mm (D30), 2.5 mm (D25), 2 mm (D20), and 1.5 mm (D15). Figure 13b illustrates the evolution characteristics of time-varying fracture widths and the distribution of crack patterns on the 24th day of corrosion age for various bar diameters. The analysis indicates that rebar diameter significantly influences the concrete surface’s cracking time. When the rebar diameter decreases, the commencement of cracking occurs sooner, and the ultimate crack width tends to reduce.

### 4.4. Cracking Patterns for Multiple Rebar Configurations

**Figure 14:**
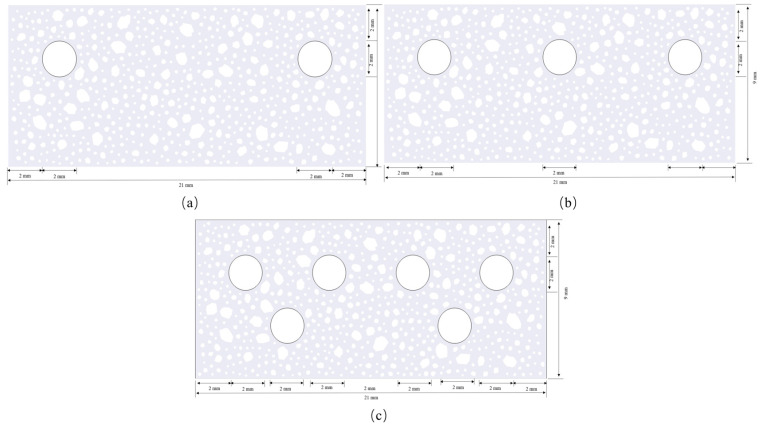
Reinforcement arrangement (**a**) double-bar; (**b**) triple-bar; (**c**) six-bar.

The developed multi-field coupled phase field model examines the influence of reinforcing configuration and interaction on the cracking behavior of concrete. The simulation investigation of concrete specimens, with a cross-section of 21 mm × 9 mm, was conducted using varying quantities of reinforcing bars, namely double-bar (Figure 14a), triple-bar (Figure 14b), and six-bar (Figure 14c) configurations. The model parameters adhere to the A1 model validated in the preceding section.

**Figure 15:**
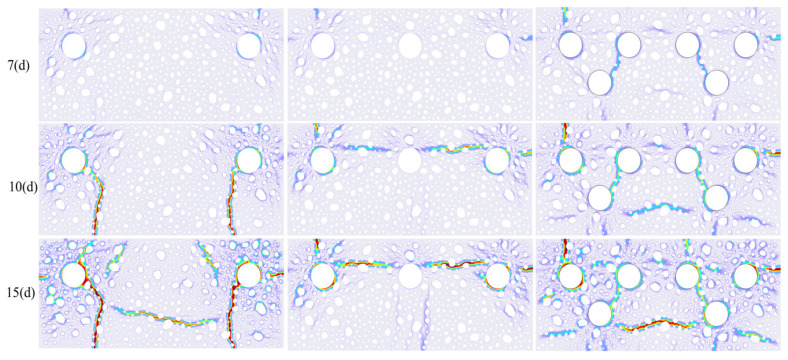
Crack patterns of different steel bar arrangements; d represents time.

**Figure 16:**
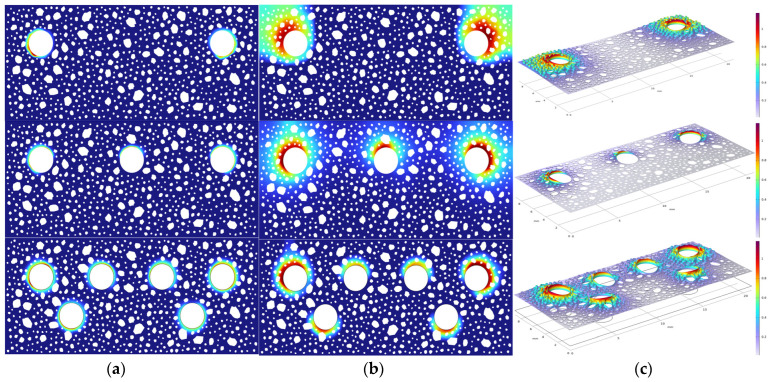
(**a**) Ferrous concentration; (**b**) Ferric concentration; (**c**) Precipitation saturation.

The cracking pattern shown in Figure 15 indicates that with a net spacing of 13 mm between reinforcement bars in a double-bar configuration, the reduced chloride ion transport path—attributable to the synergistic thickness of the protective layer and the distribution of aggregates in the corner region—facilitates the premature onset of electrolytic corrosion on the surface of the reinforcement bars in this area, resulting in considerable spalling damage in the concrete corners. By integrating the precipitation saturation cloud depicted in Figure 16c with the concentration distributions of Fe^2+^ and Fe^3+^ illustrated in Figure 16a,b on day 15, it is evident that both ferrous (Fe^2+^) and ferric (Fe^3+^) ions surrounding the corner reinforcement bars exhibited significant enrichment. The continuous accumulation of corrosion products resulted in the formation of downward-extending main cracks, which subsequently developed into penetrating cracks in the lower middle part of the specimen.

This development is likely due to the size effect of the model and the peculiar nature of the phase field approach. The cracking pattern is consistent with the typical characteristics of rust expansion cracking in actual reinforced concrete structures [56,57].

It is important to mention that the simulations revealed substantial asymmetric cracking behavior. Specifically, the middle section of the concrete protective layer was characterized by a synergistic development of mechanical bridging of aggregate particles (aggregate bridging) and parallel cracks. The right-most bar was dominated by horizontal cracks, while a distinct pattern of vertical cracks developed around the left-most bar (Figure 15).

**Figure 17:**
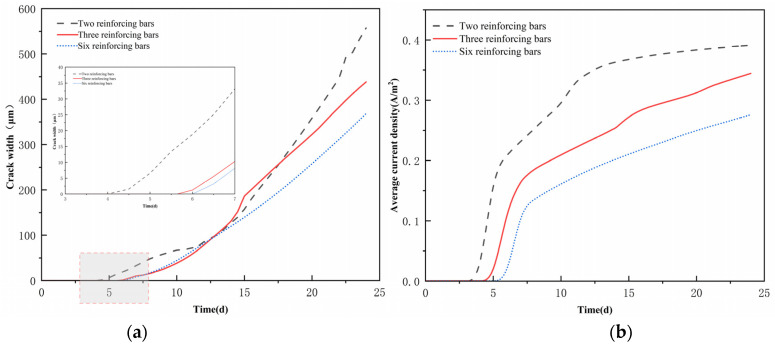
(**a**) Crack widths under different steel bar configurations; (**b**) The average current density under different steel bar configurations.

The corrosion process is profoundly and intricately affected by the alteration in the number of reinforcement rods. The number of reinforcement bars in the concrete pore solution is essentially equivalent to expanding the effective surface area of the electrode reaction, and the effective activation area grows linearly. This significantly enhances the kinetic process of the cathodic oxygen reduction reaction, where oxygen acts as the key cathodic reaction in the electrochemical system. Reinforcement bars constitute a complex electrochemical system. In this electrochemical system, oxygen serves as the primary reactant in the cathodic reaction. However, the electrochemical reaction of the overall reinforcement is impeded by the ineffective replenishment of oxygen, which leads to a decrease in current density as the number of reinforcements increases (Figure 17b).

The chemical energy generated by the corrosion electrochemical reaction is converted to mechanical energy through the mechanical energy conversion process when the net steel spacing increases, resulting in a reduction in the number of bars. This is due to the absence of adjacent reinforcement constraints and the formation of more energy dissipation, which accelerates the expansion of the width of the rust expansion cracks. Conversely, a smaller net spacing of the reinforcement and an increase in the number of reinforcements result in the coupling of the electrochemical field and stress field constraints, which prompts the formation of corrosion products. The concrete matrix stores the chemical energy generated by expansion in the form of elastic strain energy, resulting in a progressive reduction in the extent of fissures on the concrete surface (Figure 17a). In the crack width time curve of Figure 17a, an uncommon phenomenon of convergence of crack width is observed at 12.5 days. The internal oxygen content of the concrete was adequate 12.5 days ago, and the electrochemical reaction was in the oxygen activation control stage.

The crack width increased significantly as a result of the corrosion process. However, the system transitioned to the oxygen diffusion control stage after 12.5 days due to the oxygen competition effect of the electrochemical reaction between the multiple reinforcement bars in the three- and six-bar configurations. The rate of crack width growth also decreased significantly. The rate of increase in crack width experienced a substantial decrease.

**Figure 18:**
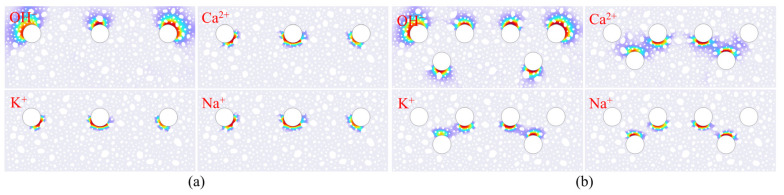
Ion distribution of different steel bar configurations: (**a**) triple-bar; (**b**) six-bar.

The distribution of Na^+^, Ca^2+^, OH^−^, and K^+^ concentrations in the surrounding pore solution after the electrochemical reaction of the rebar is shown in Figure 18 as a cloud view. The special distribution state in the corner rebar region of Figure 18b is of interest, as the concentrations of K^+^ and Na^+^ are considerably lower than those in the central rebar region. The multi-electrode coupling effect and ionic charge characteristics can be employed to elucidate this phenomenon. The corner reinforcement is situated adjacent to the concrete boundary, and its surface current density distribution is influenced by the superposition of the electric fields of the neighboring reinforcement, resulting in a non-uniform potential gradient. This localized electric field distortion deflects the electromigration paths of the monovalent cations, thereby weakening their effective transport to the cathode region. Furthermore, the migration driving force at the same electric field strength is smaller than that of divalent cations (e. g., Ca^2+^), rendering it more susceptible to the interference of secondary currents generated by neighboring electrodes. Consequently, the concentration dissipation in the corner region is exacerbated. The aforementioned ion distribution characteristics are indicative of the synergistic effects of electric field effects, ion charge properties, and multisteel coupling in the electrochemical migration process. This information serves as the foundation for the experimental validation of the refined construction of the ion transport model in the corrosion cell system.

### 4.5. Discussion

Through a multi-ionic–electrochemical–mechanical coupled phase field model, this study achieves three breakthroughs in elucidating the corrosion-induced cracking mechanism of reinforced concrete: First, the variable corrosion current density framework captures local electrochemical inhomogeneity, reducing the crack width prediction error from 29% (Korec model [31]) to 5%, thus validating the necessity of the proposed improvement. Second, the multi-physics coupling mechanism reveals the cascading effect, where chloride transport triggers non-uniform corrosion and rust expansion strain drives crack evolution, reconstructing the structural degradation path in chloride environments. Third, multi-dimensional validations demonstrate that the model can capture key characteristics of corrosion damage from initiation to propagation.

The “multi-ionic transport-microstructural damage coupling” mechanism here aligns closely with Hussain et al.’s [58] nano-silica coating study, which showed nano-silica inhibits chloride penetration via pore-filling and tortuous ion paths—consistent with our findings that concrete ITZ damage regulates multi-ionic transport and necessitates accounting for local electrochemical inhomogeneity, jointly confirming the universal “microstructure-ionic transport-corrosion kinetics” coupling. Xie et al.’s [59] identification of a “bilinear non-uniform corrosion-crack propagation correlation” in hydrogen-blended pipelines cross-validates our model’s “variable current density-driven cracking dynamics”. Despite differing materials and scenarios, both capture “corrosion-cracking cross-scale interactions”, supporting the multi-physics framework’s universality and providing a cross-domain mechanistic basis for durability design in chloride environments.

## 5. Potential Applications and Developments

Despite the effectiveness of this model in simulating localized corrosion-induced cracking behavior, the current research still has the following limitations: First, the pore structure of concrete in the model is homogenized, and the influence of the randomness of the actual pore distribution on ion transport and the diffusion of corrosion products is not fully considered. This may limit the simulation accuracy of the mass transfer process at the mesoscale. Second, the synergistic effect of carbonation and chloride erosion has not been incorporated yet, and the regulatory mechanism of the dynamic changes in temperature and humidity on corrosion kinetics is not considered. Thus, it is difficult for it to be directly applied to the simulation of structural deterioration under extreme climatic environments. Third, the self-sealing effect of the rust layer and the influence of the cumulative fatigue damage of concrete under cyclic loads on the cracking behavior are not involved. As a result, the prediction accuracy of the long-term corrosion evolution process needs to be improved.

To address the above limitations, future research can be advanced in three aspects: First, combine CT scanning technology to reconstruct the real pore morphology of concrete, and build a multi-scale coupled model of “pore-ITZ-aggregate” to improve the simulation accuracy of ion transport and the distribution of corrosion products. Second, introduce the kinetic equation of carbonation reaction, and establish a coupled framework of “carbonation-chloride erosion-mechanical damage” to expand the applicability of the model in complex environments. Third, incorporate the time-varying characteristics of the rust layer and the concrete fatigue damage model, and develop a deterioration prediction tool suitable for the whole life cycle of structures.

## 6. Conclusions

In this study, a multi-species, multi-physics phase field model is established to probe into the localized corrosion-induced cracking behavior of concrete at the microscale under chloride ion ingress. By coupling pore constraints and material inhomogeneity, the model effectively simulates the electrochemical processes encompassing metal anodic dissolution, hydration-precipitation evolution of iron ions, expansion impacts of corrosion products, and concrete cracking responses. Its reliability is validated via experimental simulations of corrosion and cracking in mortar cube specimens.

Based on parametric analyses, the mechanisms whereby parameters like interfacial transition zone (ITZ) strength, oxygen diffusion coefficient, cover thickness, and reinforcement configuration impinge on corrosion-induced cracking are systematically unveiled. The key conclusions are as follows:

(1) Through integrating a unified phase field model with continuous electrochemical reactions and multi-species transport, the localized steel dissolution process can be accurately emulated, and corrosion regions effectively differentiated. The localized corrosion current density functions as a precise gauge of reaction rate and extent.
(2) ITZ strength exerts a notable influence on the cracking mode of the concrete cover: under high ITZ strength, cracks tend to propagate along interfaces, giving rise to “aggregate boundary-type” main cracks; under low ITZ strength, cracks breach interface constraints and directly penetrate the concrete. At extremely low ITZ strength, cracks penetrating aggregates aggravate structural damage, accompanied by “multi-directional dispersed” secondary cracks and a marked increase in the maximum crack width.
(3) The oxygen diffusion coefficient modulates the corrosion reaction rates within the concrete; low oxygen concentrations impede crack width development. An increase in concrete cover thickness lessens the crack width and defers cracking initiation. A reduction in the reinforcement diameter advances the cracking onset, with the ultimate crack width exhibiting a decreasing tendency.
(4) In multi-bar systems, dense reinforcement intensifies competition in cathodic oxygen reduction reactions, engendering local oxygen-deficient zones. This shifts electrochemical reactions from activation control to mass transfer control, with the initial corrosion time extending logarithmically as the number of bars rises. The spatial superposition of rust expansion stresses from adjacent bars diminishes the stress concentration factor in the concrete matrix, resulting in a decreasing trend in the surface crack width with an increasing reinforcement ratio.

## Data Availability

The original contributions presented in this study are included in the article. Further inquiries can be directed to the corresponding author.
